# Intoxication With Endogenous Angiotensin II: A COVID-19 Hypothesis

**DOI:** 10.3389/fimmu.2020.01472

**Published:** 2020-06-19

**Authors:** Adonis Sfera, Carolina Osorio, Nyla Jafri, Eddie Lee Diaz, Jose E. Campo Maldonado

**Affiliations:** ^1^Patton State Hospital, San Bernardino, CA, United States; ^2^Department of Psychiatry, Loma Linda University, Loma Linda, CA, United States; ^3^Department of Medicine, The University of Texas Rio Grande Valley, Edinburg, TX, United States

**Keywords:** SARS-CoV-2, cellular senescence, angiotensin II, prognosis, critical illness, immune checkpoint inhibitors

## Abstract

Severe acute respiratory syndrome coronavirus 2 has spread rapidly around the globe. However, despite its high pathogenicity and transmissibility, the severity of the associated disease, COVID-19, varies widely. While the prognosis is favorable in most patients, critical illness, manifested by respiratory distress, thromboembolism, shock, and multi-organ failure, has been reported in about 5% of cases. Several studies have associated poor COVID-19 outcomes with the exhaustion of natural killer cells and cytotoxic T cells, lymphopenia, and elevated serum levels of D-dimer. In this article, we propose a common pathophysiological denominator for these negative prognostic markers, endogenous, angiotensin II toxicity. We hypothesize that, like in avian influenza, the outlook of COVID-19 is negatively correlated with the intracellular accumulation of angiotensin II promoted by the viral blockade of its degrading enzyme receptors. In this model, upregulated angiotensin II causes premature vascular senescence, leading to dysfunctional coagulation, and immunity. We further hypothesize that angiotensin II blockers and immune checkpoint inhibitors may be salutary for COVID-19 patients with critical illness by reversing both the clotting and immune defects (Graphical Abstract).

**Graphical Abstract:**
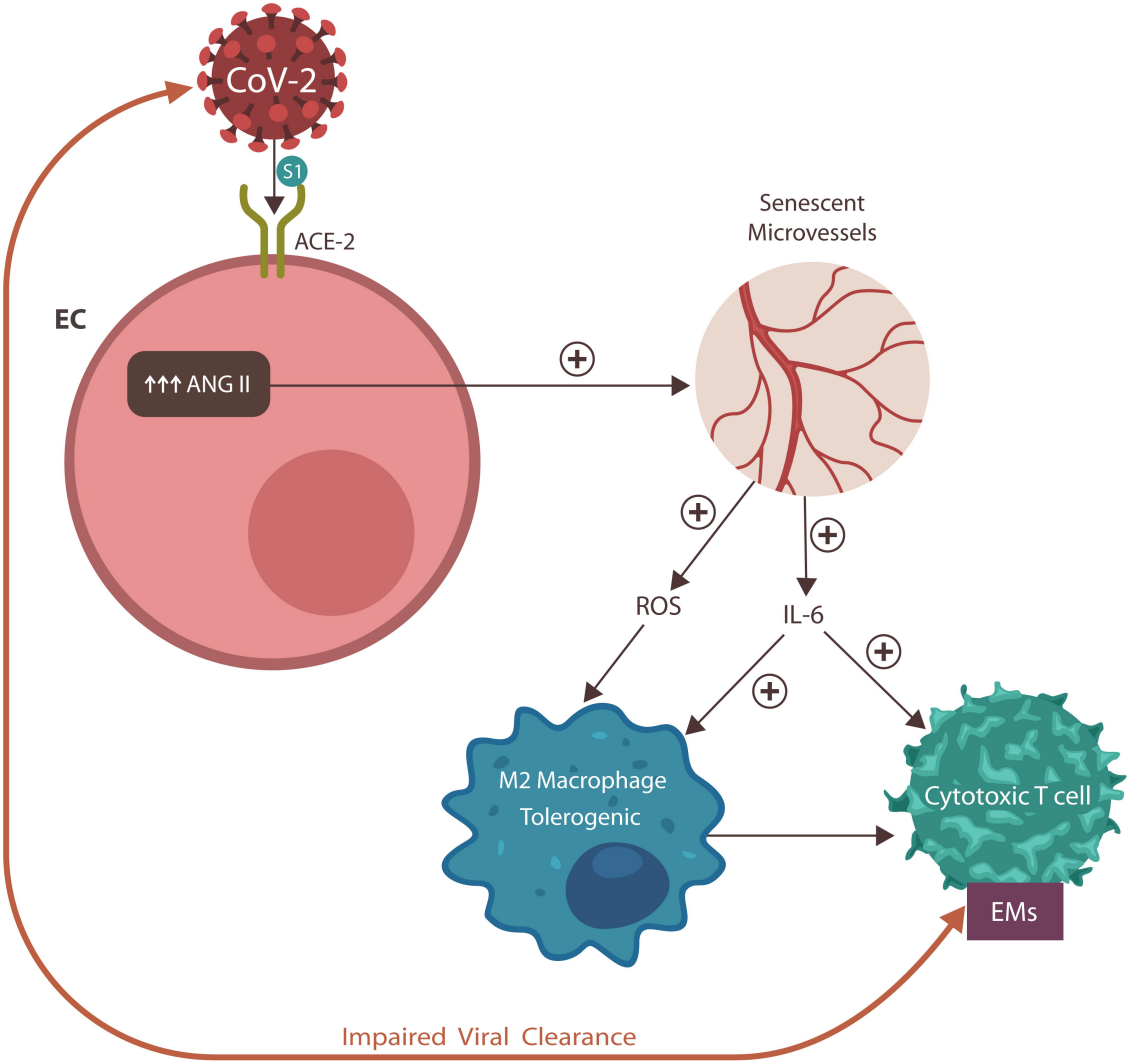
The SARS-CoV-2 virus engages the angiotensin-converting enzyme-2 (ACE-2) protein, displacing its physiological ligand. As a result, angiotensin II (ANG II) accumulates in endothelial cells (ECs), inducing vascular senescence with upregulation of interleukin-6 (IL-6) and reactive oxygen species (ROS), impairing both innate and adaptive immunity. These changes engender dysfunctional coagulation (not shown) and the expression of exhausting markers (EM). In return, these immune defects disrupt viral clearance, engendering a vicious cycle and poor COVID-19 prognosis.

## Introduction

High transmissibility, asymptomatic carriers, and the absence of herd immunity have contributed to the rapid worldwide spread of COVID-19 disease (1, 2). Although up to 50% of the affected individuals are free of clinical manifestations, about 5% of patients display serious complications, consisting of acute respiratory distress syndrome (ARDS), thromboembolism, sepsis, and multi-organ failure, often leading to death (3, 4).

COVID-19 disease is caused by the severe acute respiratory syndrome coronavirus 2 (SARS-CoV-2), which is genetically related to SARS-CoV-1, known for engendering the 2002–2003 SARS epidemic. Several studies at the time have connected this virus to severe lymphopenia, involving cytotoxic T-cells (CTCs), and natural killer (NK) cells, which are indispensable for antiviral immunity (5, 6). In addition, faulty coagulation, associated with deep venous thrombosis (DVT) and pulmonary embolism (PE), has further complicated the management of this syndrome (7). These prior findings have been replicated in relation to SARS-CoV-2 and seem to precede the development of critical illness, suggesting that defective immunity may play a major role in this disease (8–10). Indeed, as in avian influenza, the upregulation of NK cell, and CTC exhaustion markers (EMs) has been observed (11). This is somewhat surprising, as these molecules are uncommon in acute viral infections and characterize cancer and viruses associated with chronic illness, such as human immunodeficiency virus (HIV), hepatitis C virus (HCV), or cytomegalovirus (CMV) (12). In oncology, lowering EMs with immune checkpoint inhibitors (ICIs) is an established anti-tumor therapy aimed at reinvigorating host immunity, a modality with potential benefits in COVID-19 (13).

Under normal circumstances, EMs lower immune reactions to prevent autoimmunity. However, chronic inflammation can also elicit this response by prolonged stimulation of T cell receptors (TCRs) (14). Many viruses, likely including SARS-CoV-2, exploit EM pathways to avert detection. For example, SARS-CoV-2 gains access to host cells via angiotensin-converting enzyme-2 (ACE-2) associated with the renin-angiotensin system (RAS), which, aside from regulating arterial blood pressure, plays a major role in immunity (15). In this respect, SARS-CoV-2 appears to act like avian influenza viruses H5N1 and H7N9, elevating the serum levels of angiotensin II (ANG II), interleukin-6 (IL-6), and EMs (16–20).

As viral replication is more efficient in senescent cells, many viruses, including CMV and probably SARS-CoV-2, promote this phenotype in host cells to facilitate invasion (19, 21, 22). Senescent cells are characterized by proliferation arrest and a specific secretome, senescence-associated secretory phenotype (SASP). This is marked by upregulated IL-6 and reactive oxygen species (ROS), which were also detected in COVID-19 disease (23). Indeed, SARS-CoV-2 has been associated with upregulation of ANG II, a molecule previously shown to promote senescence in vascular smooth muscle cells (VSMCs) and endothelial cells (ECs) (24–26).

We hypothesize that vascular senescence-mediated upregulation of IL-6 and ROS is responsible for both coagulation and immune dysfunction. Furthermore, this pathology, evidenced by the elevated plasma levels of EMs and D-dimer, heralds a poor COVID-19 prognosis (27). We further hypothesize that ICIs and angiotensin II blockers may help critically ill COVID-19 patients by reversing the virus-induced premature vascular senescence.

## A Brief Pathophysiology of COVID-19 Disease

The SARS-CoV-2 virus gains access to host cells by engaging ACE-2 proteins, which are abundantly expressed in many tissues, including alveolar epithelial cells type II (AEC II), intestinal epithelial cells (IECs), and ECs (26, 28, 29). Interestingly, these cells function as “non-professional” antigen-presenting cells (APCs), so viral invasion directly affects their immune function. It has been established that viruses often evade detection by exploiting immunity-related host receptors. For example, the human poliovirus enters host cells via CD155, which is a receptor for T-cell immunoglobulin and ITIM domains (TIGIT) and an EM associated with functional downregulation of the CTCs and NK cells (30). Human immunodeficiency virus (HIV) upregulates EMs by maintaining a constant low-grade inflammation that repeatedly stimulates TCRs, “desensitizing” them (31). Other examples of virus-induced cellular senescence or EM upregulation are hepatitis C virus (HCV) and cytomegalovirus (CMV) (21, 32).

### ACE-2 Downregulation and Critical Illness

**Figure 1:**
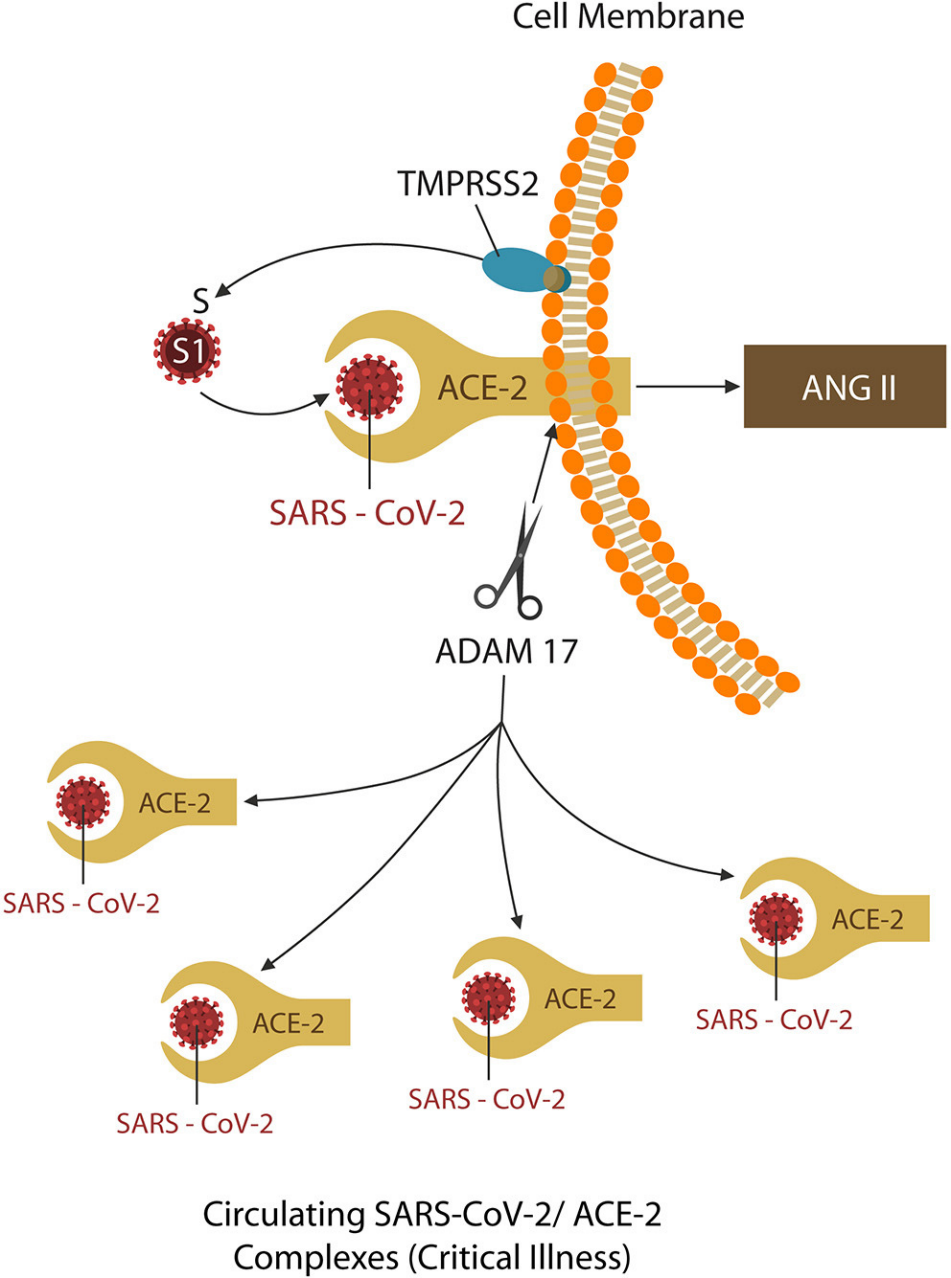
TMPRSS2 and ADAM17 are two virus-usurped host proteases. The former primes the spike (S) protein into S1, the active receptor binding site, promoting viral ingress. The latter, ADAM17, sheds the ACE-2 ectodomain, downregulating these proteins. The shed virus-ACE-2 complexes are soluble and readily spread by the circulation, causing end-organ damage, and critical illness. Some protease inhibitors may downregulate both TMPRSS2 and ADAM17, providing added therapeutic benefit for COVID-19 patients.

In SARS-CoV-1 or SARS-CoV-2 infection, unfavorable prognosis has been associated with ACE-2 downregulation (33). This is a surprising and counterintuitive finding, as fewer viral entry portals should improve the clinical outcome. However, novel studies have shown that decreased levels of ACE-2 proteins cause higher illness severity and more end-organ damage (34) (Figure 1).

On closer scrutiny, ACE-2 downregulation takes place as these proteins are shed (along with the attached virus) from the cell membranes and are spread by circulation throughout the body. This occurs as SARS-CoV-2 spike (S) protein engages ACE-2 by usurping two host proteases: type II transmembrane serine protease (TMPRSS2), which facilitates viral ingress (by cleaving the S antigen into S1, the active binding site), and ADAM17, which downregulates ACE-2 proteins (by shedding them together with the attached virus) (33–37). For this reason, the latter, responsible for COVID-19 complications and end-organ damage, may be more harmful to the host (Figure 1). Indeed, since the origination of this pandemic, the research focus has been on blocking TMPRSS2 to prevent viral entry, rather than ADAM17 inhibition to avert critical illness (26).

### SARS-CoV-2 and Cellular Senescence

**Figure 2:**
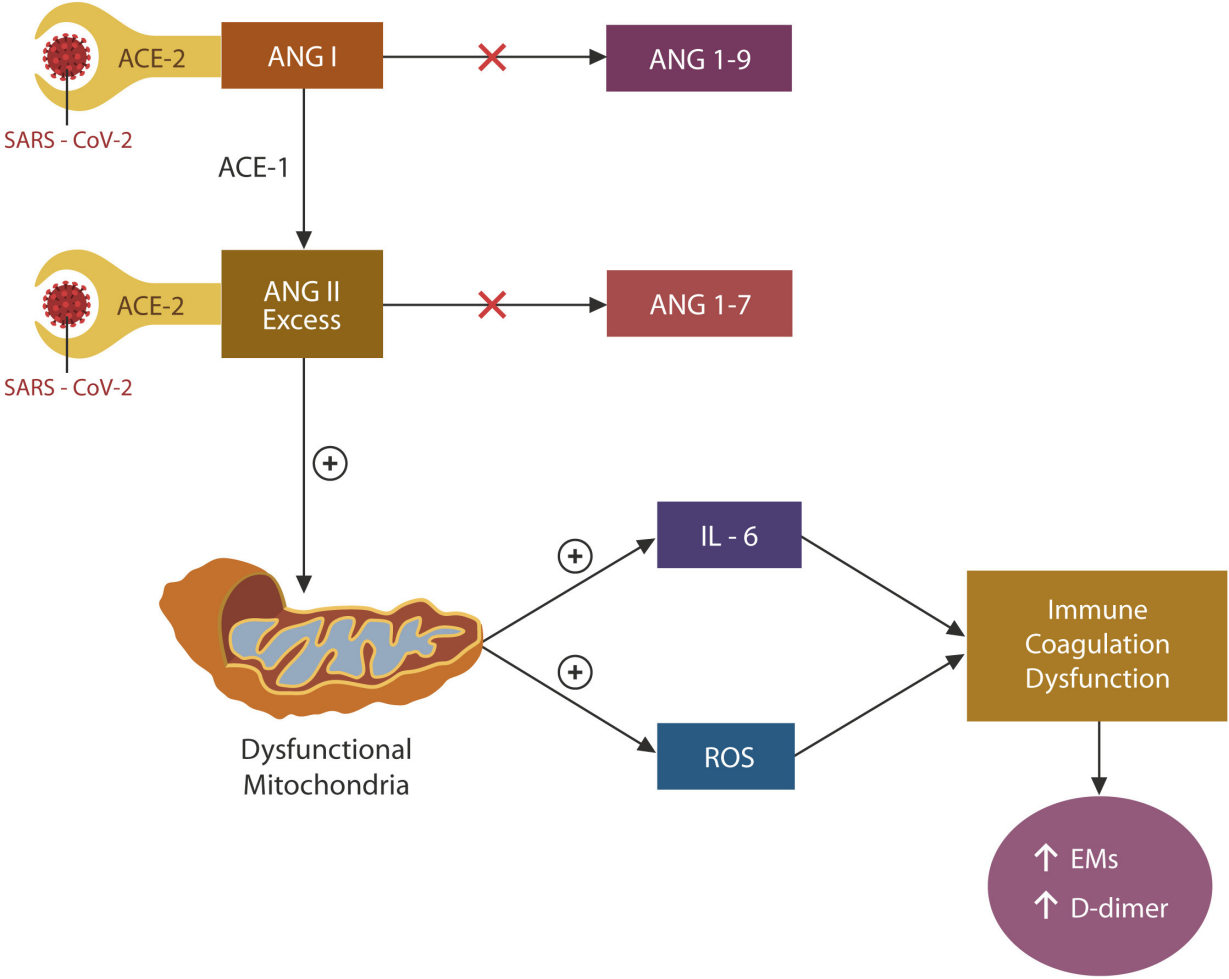
Engagement of ACE-2 by the SARS-CoV-2 virus blocks and downregulates these proteins, impairing the degradation of both ANG I and ANG II. However, since ACE-1 is not affected by the virus, ANG I conversion to ANG II continues unabated, contributing to its accumulation. ANG II excess damages mitochondria, upregulating both IL-6 and ROSs. These molecules induce EC senescence, dysfunctional immunity, and coagulation by upregulating both the exhaustion markers (EM) and D-dimer.

Under normal circumstances, ACE-2 terminates the action of angiotensin (ANG I), and ANG II by cleaving these peptides into ANG 1-9 and ANG 1-7, respectively (Figure 2). In the absence of ACE-2 (due to viral blockade and downregulation), both ANG I and ANG II accumulate. However, as ACE-1 is not engaged by the virus, the conversion of ANG I to ANG II continues unabated, leading to the unopposed accumulation of ANG II. Excess ANG II has been associated with mitochondrial oxidative damage and ROS and IL-6 upregulation, impairing both coagulation and immunity (38) (Figure 2). SARS-CoV-2 may induce vascular aging and EC senescence by two mechanisms: ADAM-17 activation and NO depletion (27, 39) (Figure 2). Indeed, preclinical studies have shown that ANG II-infused rodents demonstrated mitochondrial loss and muscle atrophy, suggesting that ANG II acts as a mitochondrial toxin (40). Taken together, SARS-CoV-2 triggers premature cellular senescence and possibly organismal aging by damaging mitochondria (41, 42).

### To ARB or Not To ARB?

A controversy involving two antihypertensive drug categories, angiotensin receptor blockers (ARBs), and angiotensin converting enzyme inhibitors (ACEi), arose as a recent paper opined that these agents might upregulate ACE-2, increasing the likelihood of viral infiltration (43). However, others have found these agents not to be harmful to COVID-19 patients and possibly to be beneficial, supporting the hypothesis presented here (44–46).

Taken together, the S1/ACE-2 attachment occupies and downregulates ACE-2 proteins, rendering them incapable of cleaving ANG II, contributing to its accumulation (Figure 2).

## Endothelial Senescence: Angiotensin II and SARS-CoV-2 Critical Illness

Under normal circumstances, ECs are facultative APCs that synthesize tissue factors and thrombin inhibitors, maintaining both coagulation and immune homeostasis (27, 47). Although SARS-CoV-2 primarily targets AEC II in the lower respiratory tract, these cells are in close proximity to the underlying endothelium, which is likely to be infected (48). Indeed, body-wide EC damage has been reported in COVID-19, suggesting that the spread of this disease outside the respiratory system is a common occurrence (49). In addition, in COVID-19, like in HIV infection, the elevated serum D-dimer levels were found to herald a higher mortality rate, linking disease severity to impaired endothelia and coagulation (50, 51). Moreover, a recent COVID-19 study found a negative correlation between D-dimer and the number of CTCs and NK cells, connecting dysfunctional coagulation with lymphopenia (52–54).

### SARS-CoV-2 and Mitochondrial Damage

**Figure 3:**
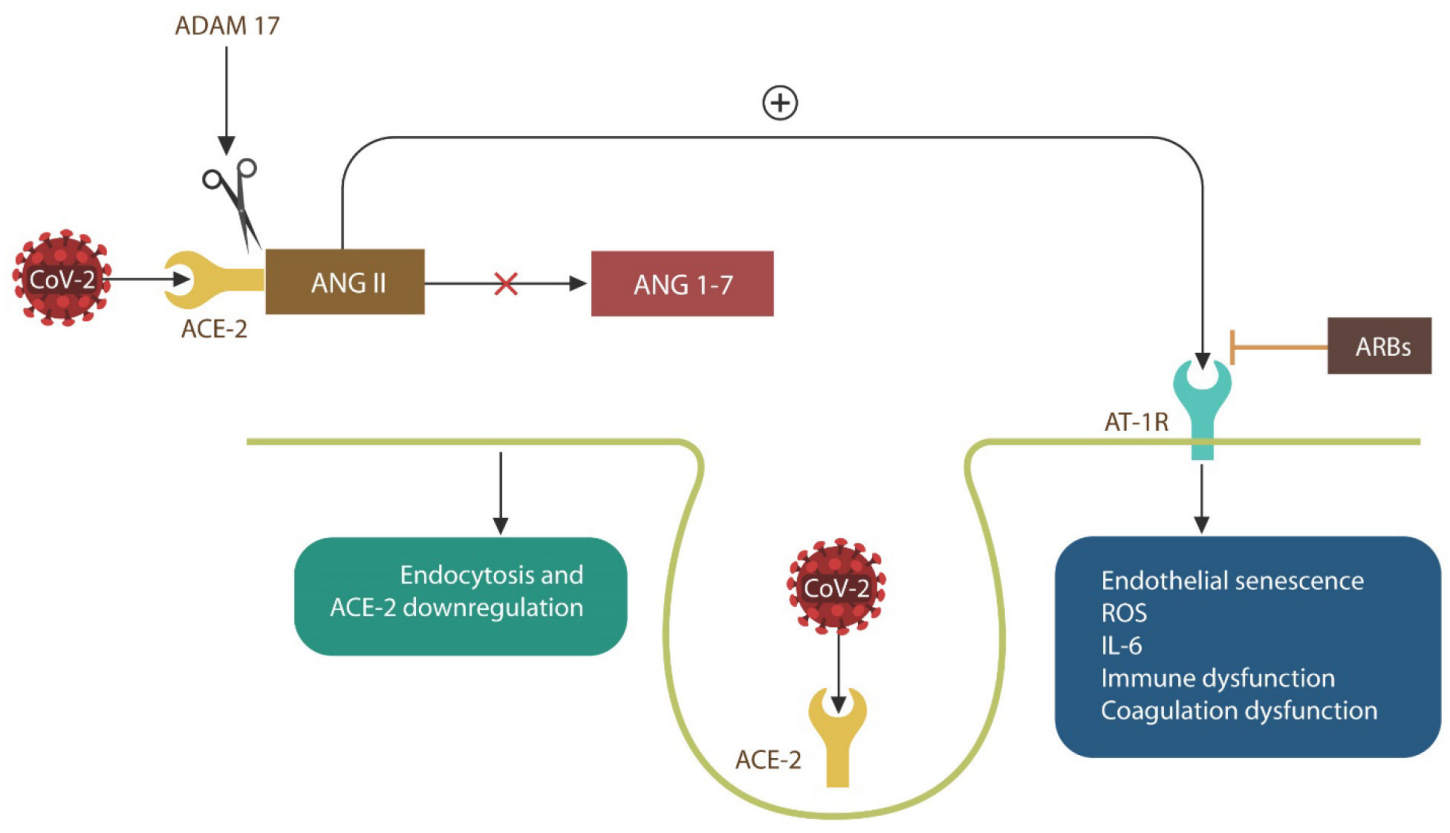
SARS-CoV-2 engagement of ACE-2 blocks ANG II breakdown into ANG 1-7, increasing intracellular ANG II. ANG II signaling via angiotensin 1 receptors (AT-1Rs) (inhibited by ARBs), induces EC senescence and upregulates IL-6 and ROS, causing immune, and coagulation dysfunction. When ACE-2 is bound by the virus, the SARS-CoV-2/ACE-2 complexes enter host cells by endocytosis. Complexes that are not endocytosed are shed by ADAM17, contributing to critical illness.

Viral replication is more effective in senescent cells, and many viruses, including influenza, have been shown to promote this phenotype in their hosts (19, 22). Indeed, the H7N9 Influenza virus induces host vascular senescence by upregulating ANG II and its signaling via AT-1Rs, causing NO depletion (19, 35, 55–59) (Figure 3). As SARS-CoV-2 is believed to utilize the same mechanism, AT-1R blockers, including losartan, are currently in COVID-19 clinical trials (NCT04335123, NCT04312009, and NCT04311177) (Figure 3).

Several viruses, including polio, HIV, and SARS-CoV-1, induce senescence in host cells by inflicting mitochondrial damage (60–62). For example, the avian influenza H5N1 virus was demonstrated to impair mitochondrial antiviral signaling (MAVS) protein, inhibiting interferon release (63, 64). Since MAVS is indispensable for NK cell and CTC maturation and metabolism, disabling these proteins translates into impaired immunity (65, 66). Aside from altering MAVS, viruses can also lower host immunity by interfering with mitochondrial metabolism. Because NK and CTCs undergo metabolic rewiring to support clonal expansion and effector function upon antigen contact, viral interference with this process impairs immune responses (67). Moreover, ROSs released by the virus-damaged mitochondria not only impair NO synthesis but also activate ADAM 17, causing EC senescence by two distinct mechanisms (27, 39, 68, 69). For this reason, ADAM17 inhibitors, deemed effective against SARS-CoV-1, should be investigated against SARS-CoV-2 (35).

### Angiotensin II, a Mitochondrial Toxin

In COVID-19 patients, elevated serum levels of ANG II were found to be directly correlated with viral load and the severity of lung injuries (70, 71). Moreover, ACE-2 downregulation has been directly linked to the critical pulmonary pathology, suggesting that unopposed ANG II acts as an endogenous toxin (72). On the other hand, a recombinant human ACE-2 (rhACE-2) was found beneficial in a small cohort of SARS-CoV-1 patients and is currently in COVID-19 clinical trials (clinical trial identifier NCT04335136) (73, 74).

Taking this evidence together, intracellular ANG II is an endogenous mitochondrial poison, causing premature endothelial senescence that damages end organs, impairing COVID-19 prognosis.

### Hypothesis- Putting It All Together

In light of the above discussion, we hypothesize the following:

1. **ANG II is a mitochondrial toxin that, under normal circumstances, is rapidly removed by ACE-2, which converts it into ANG 1-7**.
  In favor of this statement, we point to several studies showing that in the absence of hydrolyzing enzyme, ACE-2, ANG II accumulates intracellularly, inducing mitochondrial elimination or damage throughout the body endothelia (36, 75–77).
2. **ACE-2 proteins are both occupied and downregulated by the SARS-CoV-2 virus and are therefore incapable of hydrolyzing ANG II**.
  In favor of this assertion, we cite studies reporting that the SARS-CoV-2 receptor-binding domain (RBD) exhibits significantly higher affinity for ACE-2 and a higher degree of ACE-2 downregulation compared to the related SARS-CoV-1 (78, 79).
3. **The attachment of SARS-CoV-2 to ACE-2 is positively correlated with ANG II accumulation and negatively correlated with ACE-2 levels**.
  In favor of this statement are novel findings showing that ANG II serum levels are positively correlated with both the SARS-CoV-2 viral load and lung injuries (70, 71). In addition, the density of ACE-2 protein has been found to be negatively correlated with COVID-19 critical illness (72).
4. **Excess ANG II promotes premature EC senescence along with dysfunctional coagulation and immunity**.
  Several COVID-19 studies have associated poor disease prognosis with ANG II-induced endothelial dysfunction, impaired coagulation, and the overexpression of EMs (8, 27, 78, 80).
5. **SARS-CoV-2-mediated ANG II accumulation causes IL-6 and ROS upregulation, damaging the endothelia**.
  Novel studies have associated SARS-CoV-2 infection with elevation of IL-6, a cytokine that inhibits endothelial NO synthesis, causing senescence (81, 82). On the other hand, IL-6-blocking antibodies are currently in clinical trials for COVID-19 (clinical trial identifier NCT04322773). Moreover, ROSs upregulate ADAM17 and lower NO, triggering vascular aging (27, 39, 68, 69). Conversely, ROS scavengers, including camostat mesylate and anti-inflammatory/antioxidant supplements, are currently in COVID-19 clinical trials (clinical trial identifiers NCT04321096 and NCT04323228).
6. **Immune checkpoint inhibitors and ANG II blockers may help critically ill COVID-19 patients by reversing premature vascular senescence, restoring immune homeostasis**.
  We base this assertion on novel studies showing beneficial effects of rhACE-2 and ARBs, including losartan, in SARS-CoV-2 patients. Losartan and rhACE-2 clinical trials are listed above (83, 84). Moreover, cancer patients with SARS-CoV-2 who were undergoing immunotherapy were found to have a better COVID-19 prognosis than those on chemotherapy, suggesting that ICIs may be helpful against SARS-CoV-2 (85). Furthermore, the clinical trial “Personalized Immunotherapy for SARS-CoV-2 (COVID-19) Associated with Organ Dysfunction (ESCAPE)” (clinical trial identifier NCT04339712) is currently assessing the potential benefit of these agents against COVID-19.

In the remaining sections of this article, we look through the prism of this pathophysiological hypothesis, attempting to identify new target molecules, or pathways that might emerge from this model (Table 1). We also point to the neuropsychiatric manifestations of COVID-19 that, as demonstrated by prior pandemics, are often delayed and involve both movement and neurodegenerative disorders.

**Table 1:** Potential COVID-19 therapies based on the presented hypothesis.

**Drug category**	**Mechanism**	**References/clinical trials**
ADAM17 inhibitors	Blocks ACE-2 downregulation	(39)
Modified polio vaccine	Lowers CD155 and TIGIT	(86)
Aspirin	Lowers TIGIT	(87)
Anti-TIGIT antibodies	Lower TIGIT	(88)
Cariprazine	Reinvigorate immunity	None
IL-6 antibodies	Lower chronic inflammation	NCT04322773
ROS scavengers	Lower chronic inflammation	NCT04321096 and NCT04323228
BCG vaccine	Activates M1 macrophages	NCT04328441 and NCT04327206

## TIGIT: In the Eye of the “Cytokine Storms”

**Figure 4:**
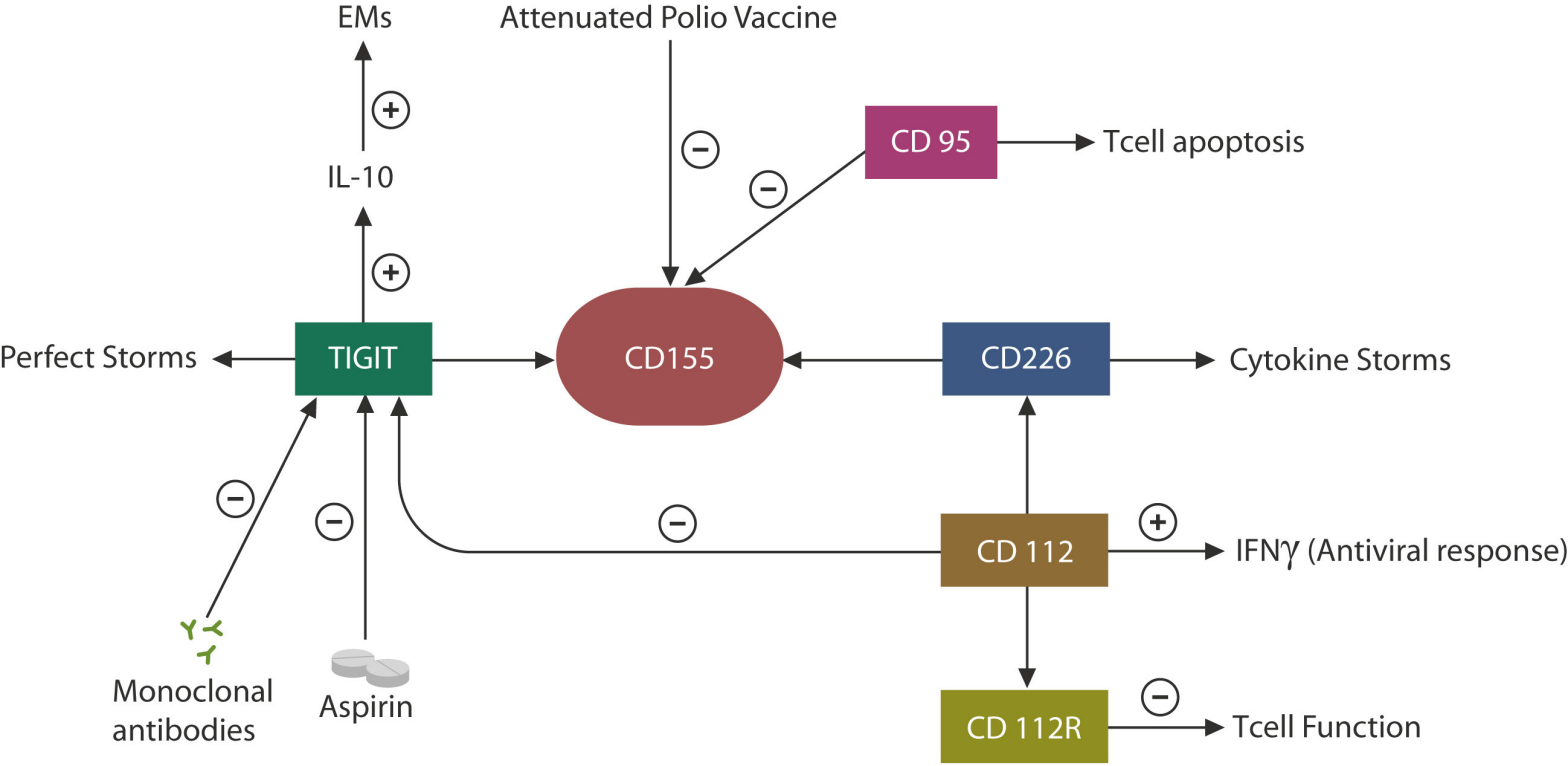
CD155 can be engaged by TIGIT, leading to immune exhaustion or by the competing molecule, CD 226, augmenting immunity. In individuals with a degree of immune senescence, TIGIT may be more likely to engage CD155, while in persons more prone to autoimmunity, CD226 may bind CD155, generating hyperinflammation, or “cytokine storms.” CD112 expression in CTCs and NK cells triggers antiviral responses by interferon release. CD112R upregulation lowers immune function as this protein, like TIGIT, functions as an EM. Viruses also exploit the CD95 pathway to induce CTC apoptosis (mimicking infection resolution). Aspirin and anti-TIGIT antibodies may decrease TIGIT, IL-10, and EMs, potentially benefiting COVID-19 patients. Attenuated polio vaccine may have similar effects by inhibiting the CD155–TIGIT axis.

COVID-19 patients may present with a wide variety of immune and inflammatory responses, ranging from hyperinflammation or “cytokine storms” to immune suppression or exhaustion (89, 90). This has raised a clinical dilemma: should immunity be augmented or lowered in COVID-19 patients? Indeed, it appears that some individuals require anti-inflammatory drugs, while others are in need of immune activators (91, 92). Along these lines, both NK cells and anti-inflammatory agents are currently in COVID-19 clinical trials, indicating that both categories may be called upon due to the fact that individual immune responses to this virus can be highly variable (NCT04375176, NCT04329650). On the one hand, the SARS-CoV-2 virus likely averts detection by inducing immune disruption, while on the other, the host may unleash excessive inflammation to limit viral infection. Since human CTCs and NK cells possess a functional RAS, the virus-induced immune impairments may be mediated by this system (93). Indeed, preclinical studies have found that ARBs, including losartan, can prevent COVID-19 pulmonary injuries, suggesting that ANG II/AT-1R signaling drives the immune defects associated with SARS-CoV-2 (70). Moreover, as the TIGIT pathway has been found to promote immune dysregulation in response to many viral infections, it is likely that SARS-CoV-2 may manipulate this EM to evade detection (10). Indeed, elevated levels of IL-10, a TIGIT-signaling cytokine, have been documented in COVID-19 patients, suggesting that SARS-CoV-2 exploits these proteins to cover its molecular signatures (Figure 4) (94).

Viruses often bind to cell membrane receptors associated with immune suppression or senescence to achieve both host cell entry and a progeny-permissive microenvironment. For example, the human poliovirus attaches to CD155, the TIGIT receptor, to upregulate this EM and inhibit host immunity (95) (Figure 4). As CD155 is associated with other immune-suppressing proteins, including CD95 and CD112 and its receptor (CD112R), it is likely that an immune inhibitory network exists around CD155 that can be exploited by viral agents to avert detection (Figure 4). For example, alpha herpesvirus targets CD112, which controls the expression of interferon gamma (IFNγ), while influenza virus induces CTC apoptosis via CD95 (96, 97) (Figure 4). Moreover, recent studies have reported that CD112R functions as a human EMs, suggesting that along with TIGIT, it may be responsible for many viral infections, including SARS-CoV-2 (98). On the other hand, TIGIT competes for CD155 binding with CD226, a receptor associated with the hyperinflammation of autoimmune disorders, suggesting the existence of a host-driven “cytokine storms” axis opposed to the virally induced immune suppressant, TIGIT (30, 99) (Figure 4). Indeed, it was recently reported that individuals expressing the CD226 G allele (which binds to CD155 with higher affinity) exhibited severe influenza symptoms, linking this gene to critical COVID-19 illness (100).

Taken together, the TIGIT–CD155–CD226 axis likely comprises a major immune switch usurped by many viruses, likely including SARS-CoV-2, to avert host detection (30, 99). As elevated serum levels of TIGIT and IL-10 have been documented in SARS-CoV-2 infection, the attenuated polio vaccine may be beneficial against COVID-19, as it inhibits CD155 and its immunosuppressive network (Figure 4).

### Older Individuals and COVID-19 Critical Illness

The COVID-19 pandemic appears to affect the elderly more than children or younger adults, suggesting that immune senescence may play a role in its pathogenesis (101–103). Since ANG II/AT-1R signaling triggers immune exhaustion, older COVID-19 patients may present with more complex immune defects engendered by the simultaneous expression of exhaustion and senescence markers (104). Indeed, novel preclinical studies have demonstrated that TIGIT knockdown can reverse premature cellular and immune aging, suggesting that downregulation of this molecule may benefit COVID-19 patients (105).

Aside from older individuals, persons with higher levels of pro-inflammatory cytokines, including those with obesity and diabetes, may be at higher risk of TIGIT overexpression and COVID-19 complications. Indeed, SARS-CoV-2 critical illness is more prevalent in individuals with these conditions, as reported by the Louisiana Department of Health Update from 3/27/2020 (106) (http://ldh.la.gov/index.cfm/newsroom/detail/5517).

**Figure 5:**
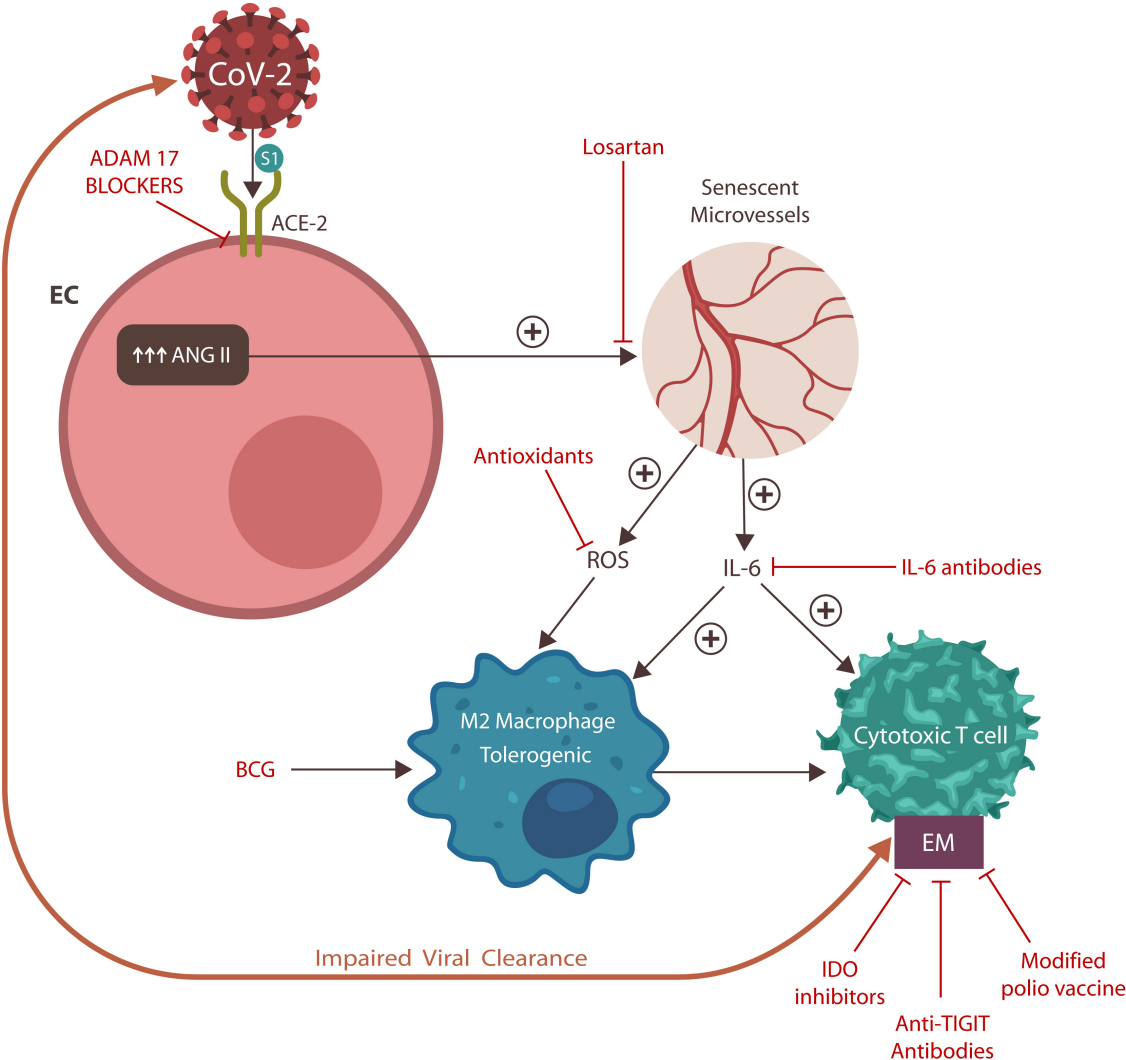
Potential anti-SARS-CoV-2 therapeutics and their action sites. ADAM17 blockers prevent ACE-2 downregulation and critical illness. Losartan blocks AT-1Rs, counteracting vascular senescence. Antioxidants and IL-6 antibodies work downstream, downregulating EM, and reinvigorating immunity. BCG activates innate immunity, improving antigen presentation to CTCs. EMs may also be lowered by cariprazine (not shown) and IDO inhibitors. Modified polio vaccine and immune checkpoint inhibitors, including anti-TIGIT antibodies, may be helpful by lowering EMs.

It is therefore possible that in individuals predisposed to autoimmunity, such as those expressing the CD226 G allele, SARS-CoV-2 may tilt the immune balance toward CD155–CD226 interaction, generating “cytokine storms.” On the other hand, in persons with preexisting immune defects, such as immune senescence, the CD155–TIGIT interaction may be enabled, engendering more profound immune deficits (by adding immune exhaustion to the previously aged CTCs and NK cells) (107, 108). Indeed, immune senescence appears to be the likely cause of the lower prevalence of autoimmune diseases and poorer response to vaccines in the elderly population (109, 110). For this reason, we surmise that the unfavorable COVID-19 prognosis is directly correlated with plasma TIGIT levels and that anti-TIGIT monoclonal antibodies could be salutary for COVID-19 patients (Figures 4, 5). Furthermore, as recombinant polio vaccines were reported to provide suitable vector systems for antigen attachment, connecting viral S protein to this vector may expedite the development of a SARS-CoV-2 vaccine (111).

## The Virus, THE RAS, and The DAS

Aside from expressing an RAS, immune cells, including dendritic cells (DC), CTCs, and NK cells, also possess a viable dopaminergic system (DAS) that plays a major role in the crosstalk between immunity and the brain (112). While the central nervous system (CNS) DAS is adequately elucidated, the role of dopamine (DA) in peripheral immunity has been less emphasized. Moreover, although a local RAS with a role in aging and cognition has previously been described in the brain, its interaction with DAS is an emerging topic in neurodegeneration, especially Parkinson's disease (PD) (113).

Nearly 40% of COVID-19 patients present with neuropsychiatric symptoms, suggesting that this virus, like many previous pandemic-related viruses, may be neurotropic (114). Indeed, delirium, seizures, impaired consciousness, and acute cerebrovascular disease have already been described in COVID-19 patients, suggesting that SARS-CoV-2 possesses the capability of altering brain functions (114, 115). Interestingly, previous studies have associated elevated D-dimer levels with strokes and delirium, indicating that, aside from the peripheral involvement, this molecule may be the herald of unfavorable neuropsychiatric outcome in COVID-19 patients (116, 118).

Aside from entering the CNS via ANG II/AT-1Rs-related senescent endothelia, SARS-CoV-2 may access the brain directly via the cribriform plate, possibly explaining the anosmia symptom described by many COVID-19 patients (119). In addition, as influenza A virus utilizes the same entry portal and lowers local immunity by inducing the nasal expression of indoleamine 2,3-dioxygenase (IDO), SARS-CoV-2 may employ a similar mechanism (120). Furthermore, IDO inhibitors, an emerging cancer therapy, may be beneficial for the neuropsychiatric manifestations of SARS-CoV-2.

Upon CNS arrival, the virus likely blocks astrocytic and neuronal ACE-2, elevating ANG II levels. In this regard, several studies have linked excessive brain ANG II to premature neuronal aging and Alzheimer's disease (AD) (120). Conversely, longevity was associated with the suppression of this molecule (121). Indeed, ARBs and ACEi were found to be protective against both PD and AD, indicating that RAS may play a key role in neurodegeneration (122–124). Moreover, in 2017, the US Food and Drug Administration approved a synthetic form of human angiotensin II, Giapreza, for the treatment of septic shock. The listed adverse effects of this compound include delirium, thrombotic events, and infection, resembling the central SARS-CoV-2 manifestations. Since ANG II accumulation may be essential for COVID-19 pathogenesis, Giapreza should probably be avoided in SARS-CoV-2-associated septic shock (113, 117, 125).

A growing body of evidence has demonstrated that DA mediates the crosstalk between immunity and the CNS, suggesting that RAS, and DAS signaling may be responsible for both peripheral and central COVID-19 manifestations. Indeed, since, at the body periphery, DA alters the activation of CTCs and NK cells, it is not surprising that DA blockers are capable of inhibiting the replication of several viruses (126–129). For example, the antiviral properties of chlorpromazine have been well-documented, as this compound protects against viral entry, preventing the exploitation of immune cells (130, 131). For these reasons, we believe that patients taking antipsychotic medications may be, at least partially, protected against COVID-19, as suggested by the emerging data on forensic inpatients (unpublished research). Moreover, as reinvigoration of CTCs can be achieved by blocking dopamine D3 receptors in DCs, selective D3 partial agonists, such as cariprazine, should be assessed for COVID-19 efficacy (132). Finally, the link between excessive DA and immune defects is further substantiated by the fact that methamphetamine (METH) users with chronically elevated DA levels often present with lymphopenia as well as CTC and NK cell dysfunction (133–135). For these reasons, METH users are probably at high risk of developing SARS-CoV-2 complications. Moreover, METH was found to augment brain ANG II/AT-1R signaling, promoting neuronal senescence, and neurocognitive deficits, further emphasizing the connection between RAS and DAS in both neurodegenerative and addictive disorders (136, 137). Conversely, ARBs are currently being tested for METH addiction, as preclinical studies have reported decreased self-administration of this stimulant in candesartan-treated rodents (138). This points to the fact that a better understanding of COVID-19 may have unintended consequences: improved treatment of addictions.

Taken together, the synergistic actions of ANG II and METH illustrate the intertwined role of RAS and DAS in both COVID-19 and substance use disorders, suggesting that candesartan may be the treatment of choice for COVID-19 in METH users.

## Conclusions

SARS-CoV-2 infection has spread around the world in a short time interval, but its prognosis is variable. Since the onset of this pandemic, there has been an overemphasis on the virus itself and less attention on host immunity.

It has been said that Nature plays a cruel game of chess in which the host and pathogen can only thrive by outmaneuvering each other. Like influenza viruses, cancer, and chronic viral infections, SARS-CoV-2 evades detection by disguising itself as an ACE-2 ligand. The host responds by mobilizing its innate and adaptive immunity to eliminate the virus, but the latter proceeds to downregulate host immune defenses by augmenting EMs. In a desperate move, the host unleashes “cytokine storms” to reinvigorate its suppressed immune cells and overcome the virus. However, this extreme maneuver sacrifices the vulnerable individuals, such as those with chronic inflammation, damaged endothelia, and defective immunity. But Nature has rarely been fair to the weak, as their demise contributes to herd immunity. And the life-death cycles go on and on, moves and countermoves, hosts and pathogens. Indeed, it has been said that man can come up with better and better mousetraps, but Nature can always build better mice.

## Data Availability Statement

The original contributions presented in the study are included in the article/supplementary material, further inquiries can be directed to the corresponding author/s.

## Author Contributions

All authors listed have made a substantial, direct and intellectual contribution to the work, and approved it for publication.

## Conflict of Interest

The authors declare that the research was conducted in the absence of any commercial or financial relationships that could be construed as a potential conflict of interest.
